# Transactions of the American Medical Association

**Published:** 1853-03

**Authors:** 


Art. VII.—*Transactions of the American Medical Association; Vol. V.* Philadelphia, 1852: pp. 939: 8vo.

This is a very large, and to every American physician must prove an exceedingly interesting volume, while to the medical inquirer everywhere, the facts relating to the epidemics of our country, and the original observations contained in some of the essays will be acceptable. Its matter is varied; the papers are from the pens of men who have devoted special attention to the subjects, and we have no hesitation in saying that as a whole it is worthy of the American medical profession. That the reader may see what the volume embraces, we subjoin the table of contents:

Minutes of the Fifth Annual Meeting of the American Medical Association; Report of the Committee of Publication and of the Treasurer; Report of the Committee on Amendments to the Constitution, &c.; Report of a minority of the Committee on Change of Representation; Report of the Special Committee on proposed Amendments to the Constitution.

*Prize Essay.*—On Variations of Pitch in Percussion and Respiratory Sounds, and their Application to Physical Diagnosis. By Austin Flint, M. D., of Buffalo, N. Y.

*Reports of Committees on Special Scientific Subjects.*—On the Blending and conversion of Types in Fever: By Samuel Henry Dickson, M. D., of Charleston, S. C.; On the Action of Water on Lead Pipes, and the Diseases proceeding from it: by Horatio Adams, M. D., of Waltham, Mass.; Permanent cure of Reducible Hernia: by George Hayward, M. D., of Boston, Mass.; Water; its Topical Uses in Surgery: by Charles A. Pope, M. D., of St. Louis, Mo.

*Reports on Epidemics.*—Report on the Epidemic Diseases of New England and New York; Report on the Epidemic Diseases which prevailed in 1851, in New Jersey, Pennsylvania, Delaware and Maryland; Report on the Epidemics in New Jersey; Report on the Epidemics of Pennsylvania; Report on the Epidemic Diseases of Delaware; Report on the Epidemic Diseases of Maryland; Report of the Committee on the Epidemics of South Carolina, Florida, Georgia, and Alabama; Report on the Epidemics of South Carolina; Report on the Epidemics of Florida; Report on the Epidemics of Georgia; Report on the Epidemics of Alabama; Table containg 1036 Cases of Periodical Fever, which occurred in the practice of Wm. M. Boling, M. D., of Montgomery, Ala., in the five years, from the 1st of June, 1843, to the last day of May, 1848; Report on the Epidemics of Ohio, Indiana, and Michigan; Report on the Epidemics of Ohio; Report on the Epidemics of Indiana; Report on the Epidemics of Michigan; Report on the Eepidemics of Tennessee and Kentucky; Report on the Epidemics of Louisiana, Mississippi, Arkansas, and Texas; Jackson, Miss., its Topography, and the Diseases of 1851: by C. S. Farrar, M. D.; Houston, Texas, its Topography, and the Diseases of 1851: by Wm. McCraven, M. D.; The Diseases of Washington, Texas, for 1851, including its Topography: by T. J. Hearde, M. D.

*Report on Medical Botany.*—A Synopsis or Systematic

Catalogue of the Indigenous and Naturalized, Flowering and Filicoid (Exogens, Endogens, and Acrogens) Medicinal Plants of the United States; with their Local, Botanical, and Medical References, and a Short Account of their Medicinal Properties; being a Report of the Committee on Indigenous Medical Botany and Materia Medica for 1850–51: by A. Clapp, M. D., Chairman. Officers of the Association for 1852; List of Permanent Members.

The Prize Essay of Prof. Flint has been already submitted to our readers We have been gratified to observe how much interest it has excited, and how generally it has received the commendation of the medical press.

The first special report is by Prof. Dickson, of Charleston, and is distinguished equally by the clear, logical method which pervades it, and the elegant style in which it is written. In reference to the "blending and conversion of Types in Fever," he thus expresses himself:

We are persuaded that the truth will, on examination, be found in the following propositions: 1. That each type, or marked variety, of fever is the result of a definite cause, relevant in its properties, character, and mode of action, to the effects produced, however obscure this relevancy may be, and ill understood, in the present state of our knowledge. 2. That these causes, varying greatly in nature, must be sometimes similar, sometimes dissimilar, and sometimes contrasted or opposite in the character or mode of their efficiency. 3. That causes of different kinds may sometimes co-exist. 4. That when they resemble each other, their effects or influences are readily *blended* and mingled and interchanged, as one or the other may predominate. 5. That when dissimilar causes coexist, they may sometimes act together, but not often; may sometimes blend their influences, but not readily or freely; they may possibly supersede each other by *substitution;* but in no imaginable instance can the effect of one cause be the effect of another and dissimilar cause. This sort of transformation, the only true conversion in the logical sense, is a rational impossibility; whether we regard diseases materially and ontologically as entites, or pathologically and dynamically as mere affections of the organism arising out of precedent impressions from cusative agents. We do not deny the difficulty of distinguishing practically such substitution from conversion so called; we desire only to express our meaning precisely, in order that our views may be clearly understood.

The following distinct Types of Fever, Prof. D. considers clearly made out:

1. The periodical, including (after Bartlett), the intermittent, remittent, and congestive.
2. The continued, comprising typhoid, true typhus, simple fever, ephemera, febriculæ, British epidemic fever, relapsing fever.
3. The exanthematous; variola, scarlatina, measles, dengue.
4. Yellow fever, the "hæmagastric pestilence" of Copland, causus of Mosely, typhus icterodes of Cullen, malignant remittent of Rush.
2. Catarrhal fever, known when epidemic, as influenza.

We regret that the limited space at present at our command forbids our giving an analysis of this admirable paper. We can give only a few extracts instead. Having spoken of the intermingling of the various types of fever, continued, yellow, exanthematous, and influenza, Dr. D. goes on to say:

"We are now prepared to appreciate correctly the phenomena of apparent *conversion* of types, the second and more difficult division of our subject. The examples above given of the mingling and interfusion of symptoms of two or more forms of fever, differ among themselves in the greater predominance of one morbid influence or another in several cases. Of numerous attacks which commence in the same way, the course, history, and ultimate termination may be strikingly diverse. Invading with the ordinary symptoms of climatorial, autumnal bilious fever, one shall retain its periodical malarious character, ending as it begun, with the features of a simple remittent. Another, losing these features in a protracted course, shall grow more and more continuous, and ultimately put on all the appearances of maculated typhus, with dry, dark tongue, mouth, teeth, and gums blackened with sordes; or present the meteorism and abdominal disorder of typhoid, with intestinal ulceration shown *post-mortem;* and a a third shall sink promptly into profound collapse, dying with orange-yellow discoloration of skin and eyes, and black vomit. If we suppose these to have been all struck down by malaria in a locality infected by the coexistence of the three morbid poisons, it is easy to imagine some of them to come under the influence of ochlesis, the alleged source of typhus and typhoid, and others, strangers and predisposed, to fall victims to the obscure cause of the hæmagastric pestilence. We may correctly assume that in all these a temporary *blending* of types took place at a certain stage of their progress, under the conjoint influence of the several concurrent causes. As they advanced, however, the more intense or forcible influence would predominate; generally speaking, the malarious characteristics, periodicity especially, would disappear, being substituted or supplanted by those of the more malignant poisons; a virtual and complete *conversion of type.* In this sense, then, we believe, that conversion is not only possible but frequent, and shall proceed to adduce in proof a few further instances."

He closes his paper with the following remarks:

"If contagious diseases can be originated or generated under any contingency whatever, and no one can doubt the possibility of this occurrence, and if the matter of contagion is, as indeed it must be, a vital individuality, a self-multiplying germ, capable of indefinite reproduction, then the creation or development of this new cause must give rise to new results. A new form of disease now presents itself, which either *blends* with that which pre-existed, or supplants and *substitutes* it. If the causes of the two are connate and correlated, and in any degree similar or analogous in influence, *blending of type* takes place; the more especially if they are nearly or quite the equal in force, and circumstances do not strongly favor either at the expense of the other. But if they be markedly dissimilar or contrasted, or in any manner incompatible, or if circumstances favor the one and repress the other, then there will and must happen the subversion of the first, the weaker or least tenacious; and *conversion of type* will take place, in the only sense possible and intelligible."

The report of Dr. Adams on the diseases proceeding from lead poison is elaborate and able, and possesses great interest for communities in which lead pipes are used for conveying water. The subject is attracting much attention, and in this paper all the results that have been brought out are presented in a condensed form. The report concludes with these words:

"It is never safe to use water drawn through lead pipes, or stored in leaden cisterns for domestic purposes, and that any article of food or drink is dangerous to health which by any possibility can be impregnated with saturnine matter. It may possibly be done in some cases with impunity, but it is *impossible to predetermine* the cases of safety where so many are fraught with danger."

The Report on the Epidemics of New England and New York consists of suggestions as to the manner in which such reports may be rendered useful. One of these is, that the Committees should be continued for a series of years. The writers say:

"A Committee appointed to investigate the epidemics of any extended section of the country needs a longer time to discover, to any great extent, its reliable sources of information; and it is eminently true of such a Committee that what is ascertained in this respect in one year, would be of essential advantage in the investigations of the next. And if, therefore, it should be appointed for a series of years, the results of its researches would become from year to year more valuable; and at the conclusion of its term of service, a summary of these results could be made out, which would be of very great value to the profession. It is obvious that comparisons could be instituted between the results of different years, by a Committee acting for the whole period, which could not be made successfully by Committees newly appointed from year to year."

The force of this must be admitted, and we may expect happy results from the adoption of the plan suggested. The Committee were not able. in the time given them, to collect any materials for a medical report.

Dr. Parrish's Report on the Epidemics of New Jersey contains much interresting matter. In reference to dysentery he says:

"Experience has taught us that it is safer to treat dysentery as an irritation merely of the intestinal mucous surface, and so far as we may judge of the practice of other physicians in our State, with whom we have conferred, we believe that the most successful treatment, every where within our borders, has been such as has conformed with the pathological views herein expressed. When a treatment has been adopted consistent with the inflammatory theory the fatality has always been alarming."

"The treatment every where, with but one exception, so far as we have seen, has been nearly the same. But one account has been received which acknowledged the necessity of depletion, and in that the disease was described as mild, tenesmus being absent, and the typhoid tendency not apparent. The treatment generally relied on has been as follows; perfect rest in the recumbent posture, with large doses of opium, to control the action of the heart; elevation of the hips to relieve partially the local congestion of the rectal vessels, abstinence from food, sponging the surface with alcoholic liquors, hot fomentations over the abdomen, effervescing draughts occasionally and ice by the mouth *ab libitum*, &c. In the commencement of the disease it has generally been arrested by such a course; if treatment has been deferred till a later period, gentle stimulants have often been required. The rate of mortality has been smaller under such a course."

The Report on the Epidemics of Pennsylvania and Delaware embraces communications from several physicians; one of which, by Dr. Condie, giving a summary of the diseases of Philadelphia for the past year, contains many valuable statistics. Dysentery was one of the prevalent diseases of the year, and the anodyne plan of treatment, we perceive, was the one which found most favor.

Dr. Carpenter communicates an important fact to the Committee. He says, that "in every obstetrical case which he has attended for two or three months past, there have been symptoms, more or less violent, of puerperal fever." Is it not questionable whether a practitioner ought to continue to attend obstetrical cases after he finds that puerperal fever is so generally the consequence? Has he not good grounds to fear that he communicates the disease to his obstetrical patients? We think this question is one which physicians ought gravely to consider. If, as there is much evidence for believing, the poison of puerperal fever be portable, and women receive the disease from their medical attendants, it surely would be well for accoucheurs who have attended one case of the disease, to abstain, for some time, from obstetrical practice.

In these reports many anomalous forms of disease are described, and to the practitioner of the country, who is in the way to meet with such cases, they must prove exceedingly interesting.

Dr. D. J. Cain's Report on the Epidemics of South Carolina is full and instructive. He mentions among the epidemics of the year an affection which he styles "*Brow Ague*," and which is thus described:

"The paroxysm usually came on between 10 and 12 o'clock in the day, with a dull aching pain, generally over one eye, but sometimes over both eyes, evidently seated in the frontal sinus. The pain gradually increased in severity until the patient was almost distracted, agonized; it then as gradually abated and almost ceased about daylight; from that time until the hour of access of the paroxysm the patient was comparatively free from pain. During the paroxysm, or more properly speaking, exacerbation, there was a free secretion of tears, but in no instance was there observed a discharge of pus. The duration of the disease was one, two, or three septenary periods, the paroxysms recurring with great regularity. The local application of chloroform gave, in most of the cases, temporary relief; but no mode of treatment, including that with quinine and bebeerine, seemed to have any effect in cutting short the disease."

Cases of this complaint have been frequent in this city during the last month, and have sometimes proved very refractory. We have derived the greatest benefit from opiates.

Paronychia is enumerated among the epidemics of the year. Dr. Cain says:

"One of the most strange of the phenomena that marked the course of the past year, was the prevalence in an epidemic form, of whitlow. This epidemic was not confined to any single section of the State, but, from the information we have been able to collect, it extended over a great portion of it. In Charleston, it was observed during a greater part of the past summer, and simultaneously in Georgetown, as reported by Dr. Miller, and also in Columbia, by Dr. J. S. Crane. We have the authority of Dr. R. W. Gibbes for the statement that, in the last-mentioned town, out of forty or fifty workmen employed in a factory, more than half the number were affected. Adults were chiefly attacked; but no death occurred from it alone, as far as could be ascertained. One patient, reported by Dr. Miller, had simultaneously three on his fingers, and one on a toe. The great majority of the cases were severe, the constitutional disturbance being high, and the inflammation soon terminating in sloughing of the soft parts: in a certain proportion, the periosteum was destroyed, leaving the bones carious or necrosed, which condition necessitated their ablation. Coincidently with the prevalence of whitlow at Georgetown, Dr. Miller observed an unusual number of furuncles, abcesses, and papular and vesicular eruptions."

The Epidemics of Georgia were typhoid fever, influenza, diarrhea, and dysentery. A longer catalogue of diseases is detailed as having been prevalent in Alabama the report of which is from Dr. Boling, of that State, and, like every thing from his pen, is careful, minute, thorough and judicious. Dr. B. succeeded in drawing out many of his brethren, who have communicated to him excellent histories. The whole communication is valuable as a summary of facts. An elaborate table of 1036 cases of periodical fever, which occurred in the practice of Dr. Boling, from 1843 to 1848, inclusive, is appended to the report, and very much enhances its value.

The Report on the Epidemics of Ohio, Indiana, and Michigan, by Dr. Mendenhall, is one of the longest and most labored in the volume. He was aided in the collection of materials for his report by Dr. Pitcher, of Detroit, and Dr. Patterson, of Indianapolis. These gentlemen have laid the profession under obligations for the industry and zeal with which they entered upon their laborious task.

On behalf of our brethren we would acknoweldge our obligations to Drs. Sutton, Bell, and Bowling, for their excellent report on the epidemics of Kentucky and Tennessee ; and we should do injustice to the Committee on the epidemics of Louisiana, Mississippi, Arkansas, and Texas, if we did not award to them similar and quite as high praise. It is one of the most encouraging circumstances connected with the present state of our profession, that so many practitioners have been found disposed to devote the time and labor necessary to bring forth such reports. It is a circumstance full of promise.

The last report in the volume, and the longest and most elaborate, is one which should have appeared in the Transactions of 1851. It was written by Dr. A. Clapp, of New Albany, and forwarded to the Association, at Charleston, but failing to reach its destination in due season, it was not inserted in the volume of that year. It is a report on the *Medical Botany of the Uuited States*, and embraces every thing worth putting upon paper on that subject. It is complete, and must form a work of reference for all future inquirers and writers on the medical botany of this country. The labor bestowed upon it by its learned author has been immense, and its fulness and acuracy are worthy of all commendation.

The report of the Committees on certain proposed amendments to the Constitution refer to some questions which are likely to excite warm discussion at the approaching meeting of the Association. It is not to be disguised that there is a party in the profession who, from the beginning, have been jealous of the influence of our medical colleges, and with whom a favorite project has been reform in that direction. With them, the end and aim of the Association is to legislate for the medical schools. As, in their estimation, all the evils that beset American medicine spring from the schools, all that is needful to purify the stream is to make the fountain pure. According to the view of this party, the body of the profession and the medical colleges are in a state of antagonism. Thus, Drs. Hays and Storer, in support of their proposition to reduce the representation of these institutions, urge this argument: "If the colleges were to make a preconcerted, united effort, they could, to a very considerable extent, control the action of the Association, and thus sacrifice the interests of 30,000 practitioners of the United States, for the benefit of about 200 professors."

Now, we confess our inability to see any force in this. We cannot understand how it can ever become the policy of the schools to "sacrifice the interests of the practitioners." Their interests, it appears to us, are one. Whatever affects the profession bears upon the schools, and whatever elevates or injures the schools must be felt by the profession. We have always been opposed to these sectarian views. We regard the schools and the practitioners as forming one profession, and cannot see how it can ever become their interest to make war upon each other. No school can prosper without the countenance and support of private physicians. The moment it places itself in an antagonistic attitude to its patrons, it has committed suicide.

We do not know, if we were a member of the Association, that we should vote against the proposed amendment — to give each medical college one delegate instead of two ; it is to the reason urged for the measure that we object. Perhaps one delegate would be a fair ratio for the schools ; it would be easy to determine that by the proportion of professors to practitioners in the former meetings. But let us have a harmonious, a homogeneous body, if possible. Let us keep down, if we can, the bttierness which party lines are sure to engender. It is to be hoped that it is as representatives of the profession, not of schools, that medical professors go to the Association. We are sure he must appear a most unworthy representative who does not act for the profession, the whole profession of his country.

The meeting of the Association soon to take place in New York, promises to be the largest yet held, and will be one of uncommon interest, not only to the delegates who may attend, but to the whole profession. These questions respecting amendments to the Constitution, we hope, will then be finally disposed of, that the Association may address itself with undivided attention to the advancement of medical science.

